# Antimicrobial resistance in clinical *Escherichia coli* isolates from poultry and livestock, China

**DOI:** 10.1371/journal.pone.0185326

**Published:** 2017-09-21

**Authors:** Afrah Kamal Yassin, Jiansen Gong, Patrick Kelly, Guangwu Lu, Luca Guardabassi, Lanjing Wei, Xiangan Han, Haixiang Qiu, Stuart Price, Darong Cheng, Chengming Wang

**Affiliations:** 1 Jiangsu Co-Innovation Center for Prevention and Control of Important Animal Infectious Diseases and Zoonoses; Yangzhou University College of Veterinary Medicine, Yangzhou, Jiangsu, PR China; 2 Department of Food Hygiene and safety, Faculty of Public and Environmental Health, Khartoum University, Khartoum, Sudan; 3 Poultry Institute, Chinese Academy of Agricultural Sciences, Yangzhou, Jiangsu, China; 4 Department of Biomedical Sciences, Ross University School of Veterinary Medicine, Basseterre, St. Kitts, West Indies; 5 Department of Diagnostic Medicine, Kansas State University, Manhattan, KS, United States of America; 6 Shanghai Veterinary Research Institute, Chinese Academy of Agricultural Sciences Shanghai, China; 7 Department of Pathobiology, College of Veterinary Medicine, Auburn University, Auburn, AL, United States of America; Kansas State University, UNITED STATES

## Abstract

Poultry and livestock are the most important reservoirs for pathogenic *Escherichia coli* and use of antimicrobials in animal farming is considered the most important factor promoting the emergence, selection and dissemination of antimicrobial-resistant microorganisms. The aim of our study was to investigate antimicrobial resistance in *E*. *coli* isolated from food animals in Jiangsu, China. The disc diffusion method was used to determine susceptibility to 18 antimicrobial agents in 862 clinical isolates collected from chickens, ducks, pigs, and cows between 2004 and 2012. Overall, 94% of the isolates showed resistance to at least one drug with 83% being resistance to at least three different classes of antimicrobials. The isolates from the different species were most commonly resistant to tetracycline, nalidixic acid, sulfamethoxazole, trimethoprim/sulfamethoxazole and ampicillin, and showed increasing resistance to amikacin, aztreonam, ceftazidime, cefotaxime, chloramphenicol, ciprofloxacin. They were least resistant to amoxicillin/clavulanic acid (3.4%) and ertapenem (0.2%). MDR was most common in isolates from ducks (44/44, 100%), followed by chickens (568/644, 88.2%), pigs (93/113, 82.3%) and cows (13/61, 21.3%). Our finding that clinical *E*. *coli* isolates from poultry and livestock are commonly resistant to multiple antibiotics should alert public health and veterinary authorities to limit and rationalize antimicrobial use in China.

## Introduction

*Escherichia coli* is a common commensal organism in people and animals with certain strains being pathogenic and causing conditions including gastroenteritis, cystitis, meningitis, peritonitis, and septicemia. *E*. *coli* strains are considered to be excellent indicators of antimicrobial resistance because they are part of the normal microbiota of people and animals, and also occur in the environment [1].

Antimicrobial resistance is a global health concern in both human and veterinary medicine [2–4] where antimicrobial agents have been used widely for treating bacterial diseases [5]. The use and misuse of antimicrobial agents has led to the development of resistance which is threatening their effectiveness in the treatment and prevention of bacterial infections [6].

China is the largest user of antibiotics in the world with 162,000 tons used in 2013 [7], about 10 times more than used in the USA [8]. Mostly, these antibiotics are used in the livestock and poultry industries where there are few regulations controlling their use [9]. Recent studies based on antibiotic resistance genes (ARGs) profiling in manure, soil and water from 5 swine farms, 6 chicken farms and 5 cattle farms in southeastern China have shown that antimicrobial resistance was widespread in animals and the environment, with 22 ARG profiles representing resistance to five major classes of antibiotics (tetracyclines, sulfonamides, quinolones, aminoglycosides, and macrolides) identified in sixteen farms [8].

There is growing awareness of the importance of antimicrobial resistance in China and an increasing number of reports on the situation in people and animals [10]. Such information provides a meaningful foundation that will enable a more rational approach to the prescribing and use of antimicrobials in China with regulations and legislation that will help prevent the development antimicrobial resistance [10]. Most previous studies on antimicrobial resistance in *E*. *coli* isolated from animals focused on commensal isolates from feces or manure [11–13]. Currently, there is limited data on antimicrobial resistance in veterinary clinical isolates from China. Hence, we tested antimicrobial susceptibility in a large collection of *E*. *coli* isolated from diseased chickens, ducks, pigs and cows from the Jiangsu Province.

Disk susceptibility testing is the most commonly used technique in clinical microbiology laboratories to determine resistance to a wide range of antimicrobials. We therefore used this test to determine antimicrobial resistance in *E*. *coli* against 18 antimicrobials. The test can be unreliable in determining colistin resistance, causing minor errors, and other tests such as Etest, disk prediffusion and MIC are more accurate for this antibiotics [14].

## Materials and methods

### Bacterial isolates

We used 862 extra-intestinal clinical isolates of *E*. *coli* archived in the Veterinary Microbiology Laboratory of the College of Veterinary Medicine, Yangzhou University. These isolates originated from chickens (n = 644), pigs (n = 113), cows (n = 61) and ducks (n = 44) from various areas in Jiangsu Province between 2004 and 2012.

### Antimicrobial susceptibility testing

All the *E*. *coli* isolates were tested for susceptibility against 18 antimicrobial agents using the disk agar diffusion method according to the Clinical Laboratory Standard Institute guidelines [15] (CLSI, 2013). *E*. *coli* ATCC 25922 was used as a reference strain for quality control. The antibiotics disks included ampicillin (10μg), amoxicillin/clavulanic acid (30μg), cefotaxime (30μg), ceftazidime (30μg), ceftriaxone (30μg), ertapenem (10μg), aztreonam (30μg), streptomycin (10μg), gentamicin (10μg), amikacin (30μg), tetracycline (30μg), ciprofloxacin (5μg), nalidixic acid (30μg), enrofloxacin (5μg), sulfamethoxazole (300μg), trimethoprim/sulfamethoxazole (1.25/23.75 μg), chloramphenicol (30μg) and nitrofurantoin (300μg). A multidrug-resistant (MDR) strain was defined as one which was resistant to at least three different classes of antimicrobials [16].

### Statistical analysis

The antibacterial resistance rates in different animals species was compared using the Chi-squared Test (StatSoft, Inc., Oklahoma, USA). Differences at P≤0.05 were considered significant.

## Results

**Fig 1:**
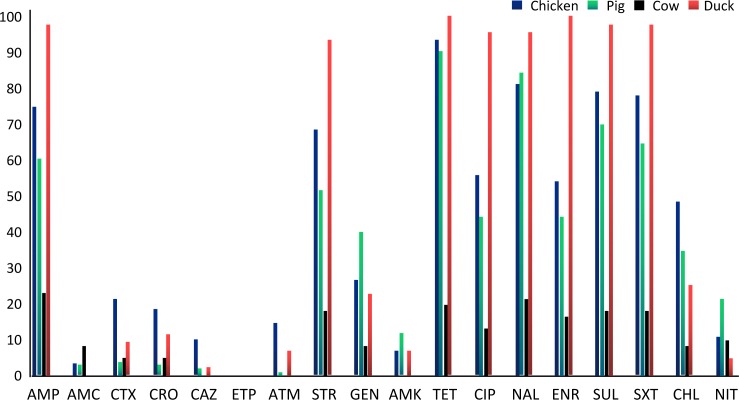
Antimicrobial resistance to 18 antibiotics of *E*. *coli* isolates from different food animal sources. The columns denote the percentages of resistant isolates for individual animal species (chicken, pig, cow and duck). AMP, ampicillin; AMC, amoxicillin/ clavulanic acid; CTX, cefotaxime; CRO, ceftriaxone; CAZ, ceftazidime; ETP, ertapenem; ATM, anaztreonam STR, streptomycin; GEN, gentamicin; AMK, amikacin; TET, tetracycline; CIP, ciprofloxacin; NAL, nalidixic acid; ENR, enrofloxacin; SUL, sulfisoxazole; SXT, trimethoprim/sulfamethoxazole; CHL, chloramphenicol; NIT, nitrofurantoin.

Overall, the antibacterial resistance rates in extra-intestinal clinical isolates of *E*. *coli* from chickens, pigs and ducks were significantly higher than in cows (P≤10^−4^). Ninety-four percent of the *E*. *coli* isolates (810/ 862) showed resistance to at least one antimicrobial, but none were resistant to all of the 18 antimicrobials tested (Table 1, Fig 1). The highest rates of resistance (>75%) were found with tetracycline, nalidixic acid, sulfamethoxazole, ampicillin, enrofloxacin and trimethoprim-sulfamethoxazole. There were increasing in resistance to amikacin, aztreonam, ceftazidime, cefotaxime, chloramphenicol, ciprofloxacin.

**Table 1:** Percentage of antimicrobial resistance among *Escherichia coli* isolates.

Antibiotics	Isolates from chickens (n = 644)			Isolates from pigs (n = 113)			Isolates from cows (n = 61)			Isolates from ducks (n = 44)		
	R	I	S	R	I	S	R	I	S	R	I	S
**Amikacin**	43(6.7%)	18(2.8%)	583(90.5%)	13(11.5%)	0%	100(88.5%)	0%	0%	61(100%)	3 (6.8%)	2 (4.6%)	39 (88.6%)
**Gentamicin**	170(26.4%)	444(68.9%)	30(4.7%)	45(39.8%)	44(38.9%)	24(21.3%)	5(8.2%)	47(77.0%)	9(14.8%)	10 (22.7%)	34 (77.3%)	0%
**Streptomycin**	440(68.3%)	163(25.3%)	41(6.4%)	58(51.3%)	41(36.3%)	14(12.4%)	11(18.0%)	23(37.7%)	27(44.3%)	41 (93.2%)	3 (6.8%)	0%
**Ertapenem**	1(0.2%)	2(0.3%)	641(99.5%)	0%	0%	113(100%)	0%	0%	61(100%)	0%	0%	44 (100%)
**Cefotaxime**	137(21.3%)	11(1.7%)	496(77.0%)	4(3.6%)	0%	109(96.4%)	3(4.9%)	0%	58(95.1%)	4 (9.1%)	1 (2.3%)	39 (88.6%)
**Ceftazidime**	63(9.8%)	28(4.3%)	553(85.9%)	2(1.8%)	3(2.7%)	108(95.5%)	0%	0%	61(100%)	1 (2.3%)	1 (2.3%)	42 (95.4%)
**Ceftriaxone**	117(18.2%)	9(1.4%)	518(80.4%)	3(2.7%)	1(0.9%)	109(96.4%)	3(4.9%)	0%	58(95.1%)	5(11.4%)	0%	39 (88.6%)
**Aztreonam**	94(14.6%)	17(2.6%)	533(82.8%)	1(0.9%)	3(2.7%)	109(96.4%)	0%	0%	61(100%)	3 (6.8%)	1 (2.3%)	40 (90.9%)
**Nitrofurantoin**	68(10.6%)	99(15.4%)	477(74.0%)	24(21.2%)	34(30.1%)	55(48.7%)	6(9.8%)	1(1.6%)	54(88.5%)	2 (4.6%)	4 (9%)	38 (86.4%)
**Amoxicillin/ Clavulanic acid**	21(3.3%)	75(11.6%)	548(85.1%)	3(2.7%)	8(7.1%)	102(90.2%)	5(8.2%)	0%	56(91.8%)	0%	1 (2.3%)	43 (97.7%)
**Ampicillin**	482(74.8%)	50(7.8%)	112(17.4%)	68(60.2%)	5(4.4%)	40(35.4%)	14(23.0%)	8(13.1%)	39(63.9%)	43 (97.7%)	0%	1 (2.3%)
**Ciprofloxacin**	358(55.6%)	40(6.2%)	246(38.2%)	50(44.2%)	6(5.3%)	57(50.5%)	8(13.1%)	2(3.3%)	51(83.6%)	42 (95.4%)	1 (2.3%)	1 (2.3%)
**Nalidixic acid**	523(81.2%)	6(0.9%)	115(17.9%)	95(84.1%)	2(1.8%)	16(14.1%)	13(21.3%)	1(1.6%)	47(77.1%)	42 (95.4%)	1 (2.3%)	1 (2.3%)
**Enrofloxacin**	347(53.9%)	69(10.7%)	228(35.4%)	50(44.2%)	15(13.3%)	48(42.5%)	10(16.4%)	1(1.6%)	50(82.0%)	44 (100%)	0%	0%
**Sulfamethoxazole**	508(78.9%)	1(0.2%)	135(20.9%)	79(69.9%)	0%	34(30.1%)	11(18.0%)	0%	50(82.0%)	43 (97.7%)	0%	1 (2.3%)
**Trimethoprim/ sulfamethoxazole**	501(77.8%)	1(0.2%)	142(20.0%)	73(64.6%)	1(0.9%)	39(34.5%)	11(18.0%)	0%	50(82.0%)	43 (97.7%)	0%	1 (2.3%)
**Tetracycline**	602(93.5%)	2(0.3%)	40(6.2%)	102(90.2%)	0%	11(9.8%)	12(19.7%)	0%	49(80.3%)	44 (100%)	0%	0%
**Chloramphenicol**	311(48.3%)	33(5.1%)	300(46.6%)	39(34.5%)	8(7.1%)	66(58.4%)	5(8.2%)	0%	56(91.8%)	11 (25%)	0%	33 (75%)

R = resistant; I = intermediate; S = susceptible

Amongst the antimicrobials with moderate rates of resistance, gentamicin was the most prevalent in all four animal species (chicken, 68.9%; pig, 38.9%; cow, 77.0%; duck 77.3%), followed by streptomycin (chickens, 25.3%; pigs, 36.3%; cows, 37.7%; ducks, 6.8%) (Table 1). The *E*. *coli* isolates from all four animal species were most susceptible to ertapenem (0.2% chicken; 0% in pigs, cows and ducks) and amoxicillin/clavulanic acid (chickens, 3.3%; pigs, 2.7%; cows, 8.2%; ducks, 0%).

**Fig 2:**
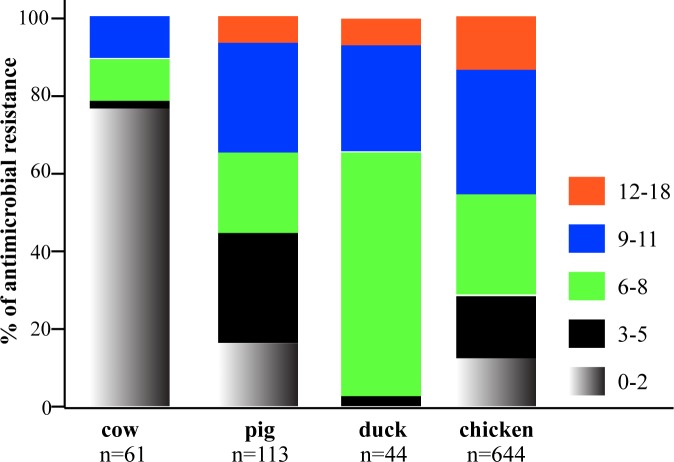
Antibacterial resistance patterns of *Escherichia coli* isolates. **A.** Percentages of *E*. *coli* isolates from chickens, pigs, cows and ducks were resistant to 0–2, 3–5, 6–8, 9–11 and 12–18 antibacterial drugs, respectively. While 78.7% (48/61) of cow isolates showed resistance to 0–2 drugs, the pig isolates showed similar resistances to 3–5 drug (27.4%, 31/113), 6–8 drugs (23.0%, 26/113), and 9–11 drugs (24.8%, 28/113). In ducks, most isolates were resistant to 6–8 drugs (61.4%, 27/44) and 9–11 drugs (31.8%, 14/44). The worst bacterial resistance was seen in chickens, and 71.6% of the isolates were resistant to more than 6 antibacterial drugs (6–8 drugs: 26.2%, 169/664; 10–12 drugs: 26.2%, 169/644; 12–18 drugs: 15.7%, 101/644.

Overall, 83.3% (718/862) of the isolates showed resistance to at least three antimicrobials with a significantly higher number of the isolates from ducks (44/44, 100%), chickens (568/644, 88.2%) and pigs (93/113, 82.3%) being resistant as compared to those from cows (13/61, 21.3%) (P<10^−4^). The maximum number of antimicrobials against which isolates were resistant was 16 with such isolates originating from chickens, pigs and ducks (Fig 2). Isolates from cows were resistant to fewer antimicrobials, up to 11.

## Discussion

In this study, we investigated the prevalence of antimicrobial resistance in clinical *E*. *coli* isolates from poultry and livestock in Jiangsu province. Our findings show different resistance patterns to several old antibiotics that have been commonly used in animal’s practices in China for a long time [17], mainly tetracycline, nalidixic acid, sulfamethoxazole, trimethoprim-sulfamethoxazole and ampicillin. High resistance rates to tetracycline in isolates from the four animal species we studied have also been recorded in China and other countries where the drug is widely used in treating bacterial disease and promoting feed conversion efficiency [18, 19].

In the case of the penicillins, resistance to ampicillin was most common (overall 70.4%), while resistance to amoxicillin/clavulanic acid was uncommon in all species (overall 3.4%). Other studies have also shown similar high resistance rates against ampicillin (over 70%) in *E*. *coli* from chickens in China where the ampicillin resistant rate rose from 23.1% in 1970 to 74.6% in 2003 [4, 20]. The prevalence of resistance to third-generation cephalosporins, mainly ceftazidime, ceftriaxone and cefotaxime, were relatively low in our study (7.6%, 17.1% and 14.8%) although higher rates (32.7%) were recorded in a previous study of food animals in China [19].

The quinolone antibacterial drugs have been used widely in veterinary practice in China and it was not unexpected that we found high resistance rates to most of these drugs with resistance to nalidixic acid being the second most prevalent of all the antimicrobials tested. This finding is consistent with a previous study from China in which all the clinical *E*. *coli* isolates were resistant to nalidixic acid [20]. We also found high resistance rates of over 50% to ciprofloxacin and enrofloxacin, suggesting that the main mechanisms of resistance to quinolones are chromosome-encoded, due to modifications of molecular targets (DNA gyrase and topoisomerase IV) [21].

Resistance rates amongst the aminoglycosides were highest for gentamicin (63.8%) and streptomycin (26.2%) and lowest against amikacin (6.8%) which might be because amikacin is not approved for use in food animals in China [22]. Aminoglycosides are most commonly used in pet animal practice in China where relatively higher levels of the resistance (28.5%) in *E*. *coli* isolates have been found [23]. A similar finding was observed in a study conducted in Switzerland [24].

Of particular note was our finding of high rates of resistance to chloramphenicol in all four animal species (overall 42.5%). Although chloramphenicol has been banned for use in food animals since 2002 in China, a similar high level of resistance was reported in chickens from China in 2010 (51.8%) and more than double the level found in 1993 (23.2%) [18]. There was no clear explanation for these high levels of resistance but it might be related to the use of florfenicol, a fluorinated derivative of chloramphenicol, which was approved in 1996 to treat bovine respiratory infections and thus could have been introduced into many livestock operations [25].

The high levels of resistance to the sulfonamides found in this study has also been reported from China previously [20, 26] and is not unexpected as sulfonamides have been in wide and continuous use for over 80 years with resistance already described back in 1950.

It was noteworthy that the *E*. *coli* isolates collected from cattle were less frequently resistant to the various antimicrobials we tested, a finding that has also been reported in other studies [27, 28]. One possible reason may be that antimicrobial use is lower in cattle than in other animals. Similarly, the cattle isolates had relatively lower levels of MDR compared to the very high levels found in the poultry and the pig isolates. This is consistent with the findings in a previous report [27] and a national surveillance study which showed high levels of MDR *E*. *coli* isolates in chickens (89.20%; 6,751/7,568) and pigs (90.00%; 6,806/7,562) in China [19].

The high levels of antimicrobial resistance amongst the *E*. *coli* isolates in our study is generally consistent with those reported in studies conducted in other areas of China. There is growing evidence that *E*. *coli* infections of animals and people are becoming increasingly difficult to treat in China and that guidelines and regulations are urgently needed to limit and rationalize antimicrobial use.
